# Clinical efficiency of three-port inflatable robot-assisted thoracoscopic surgery in mediastinal tumor resection

**DOI:** 10.1186/s12957-024-03357-x

**Published:** 2024-03-25

**Authors:** Hao Peng, YuanPeng He, Siqi Sheng, Maierhaba Maitiyasen, Jingfeng Li, Yvxuan Liu, Jing Chen, Xinyu Hou, Haizhu Song, Jun Yi

**Affiliations:** 1 Department of Cardiothoracic Surgery, Jinling Hospital, Affiliated Hospital of Medical School, Nanjing University, 305 East Zhongshan Road, Nanjing, Jiangsu Province PR China; 2 Department of Medical Oncology, Jinling Hospital, Affiliated Hospital of Medical School, Nanjing University, 305 East Zhongshan Road, Nanjing, Jiangsu Province PR China; 3 Department of Cardiothoracic Surgery, Jinling Hospital, Nanjing University Of Chinese Medicine, Nanjing, Jiangsu Province PR China

## Abstract

**Background:**

Aimed to assess clinical effect of three-port inflatable robot-assisted thoracoscopic surgery in mediastinal tumor resection by comparing results of the robot group with the video group.

**Methods:**

Retrospectively analyze 179 patients diagnosed with anterior mediastinal tumor from May 2017 to August 2021. Two groups were divided according to the surgical approach, including 92 cases in the RATS group and 87 cases in the VATS group. The results were analyzed between two groups with variables of age, sex, BMI, tumor size, and diagnosis. Perioperative clinical data was gathered to compare.

**Result:**

There were no significant differences between the 2 groups with regards to demographic data and clinical features. There were no significant differences inoperative time and duration of chest tube via RATS vs. VATS. The intraoperative blood loss was statistically significantly different among the RATS and VATS groups (75.9 ± 39.6 vs. 97.4 ± 35.8 ml *p* = 0.042). The postoperative stay of patients in RATS group were significantly shorter than that in VATS group (2.3 ± 1.0 vs. 3.4 ± 1.4 day *p* = 0.035),

**Conclusion:**

Three-port inflatable robot-assisted thoracoscopic surgery for mediastinal tumor is feasible and reliable it is more advantageous, and it provides the surgeon with advice on treatment choice.

**Supplementary Information:**

The online version contains supplementary material available at 10.1186/s12957-024-03357-x.

## Introduction

In the realm of thoracic afflictions, mediastinal masses comprise approximately 3% of cases [1, 2]. Among these, anterior mediastinal lesions (AML) exhibit diverse clinical and pathological characteristics. Mediastinal goiter and thymoma are predominantly situated in the anterior mediastinum [3]. The cornerstone of multimodal therapy for AML is radical resection, with the choice of surgical approach generally determined by the size and nature (benign or malignant) of the tumors [4]. The conventional open transthoracic procedure is the gold standard for AML, while video-assisted thoracoscopic surgery (VATS) has gained widespread acceptance in the past decade, aiming to minimize surgical trauma, blood loss, postoperative complications, and shorten hospital stays [5–7]. However, VATS is constrained by its two-dimensional view and reduced dexterity. With advancements in surgical instrumentation, robot-assisted thoracic surgery (RATS) offers superior dexterity and reduces surgeon fatigue. It features a surgeon-controlled stable 3D camera, an ergonomic operating position, and Endo-Wristed instruments with 7 degrees of freedom, making it particularly suitable for training novices [8–10]. Although there are only a few case reports discussing the advantages of RATS in AML, no consensus has been reached. To assess the therapeutic efficacy of RATS for AML, we conducted a retrospective study to evaluate the clinical outcomes of RATS. In this report, we delineate our surgical strategy and experience for anterior mediastinal lesions, presenting the clinical outcomes of patients who underwent surgical resection of anterior mediastinal lesions with the da Vinci robotic system at our institution.

## Materials and methods

### Patient

From May 2017 to August 2021, a total of 179 patients underwent resection of anterior mediastinal lesions at the Department of Thoracic Surgery in Nanjing Jinling Hospital. The diagnoses and clinical information were gathered by reviewing the patients’ medical records. Enhanced computed tomography scans (CT scans) were employed to pinpoint the location of the tumor. Patient inclusion criteria were as follows: (I) under 75 years of age, (II) diagnosed with resectable AML, and (III) willing to undergo RATS or VATS. Exclusion criteria consisted of: (I) tumor size larger than 8 cm, (II) poor cardiac or lung function, or severe arrhythmia. Demographic and clinical characteristics encompassed age, gender, body mass index (BMI), and underlying conditions such as hypertension, diabetes, and chronic obstructive pulmonary disease (COPD). Tumor-related data included tumor size and type. Surgical-related data encompassed total operative time, intraoperative blood loss, duration of chest tube placement, volume of drainage, postoperative hospital stay, intraoperative adverse events, and complications. This study received approval from our Institutional Review Board, and written informed consent was obtained.

### Surgical method

The surgical approach was tailored to the individual based on both tumor size and patient preference. Preoperative preparation adhered to the standard protocol for conventional anterior mediastinal surgery. Typically, the lateral approach was employed, either via the right or left side.

**Fig. 1:**
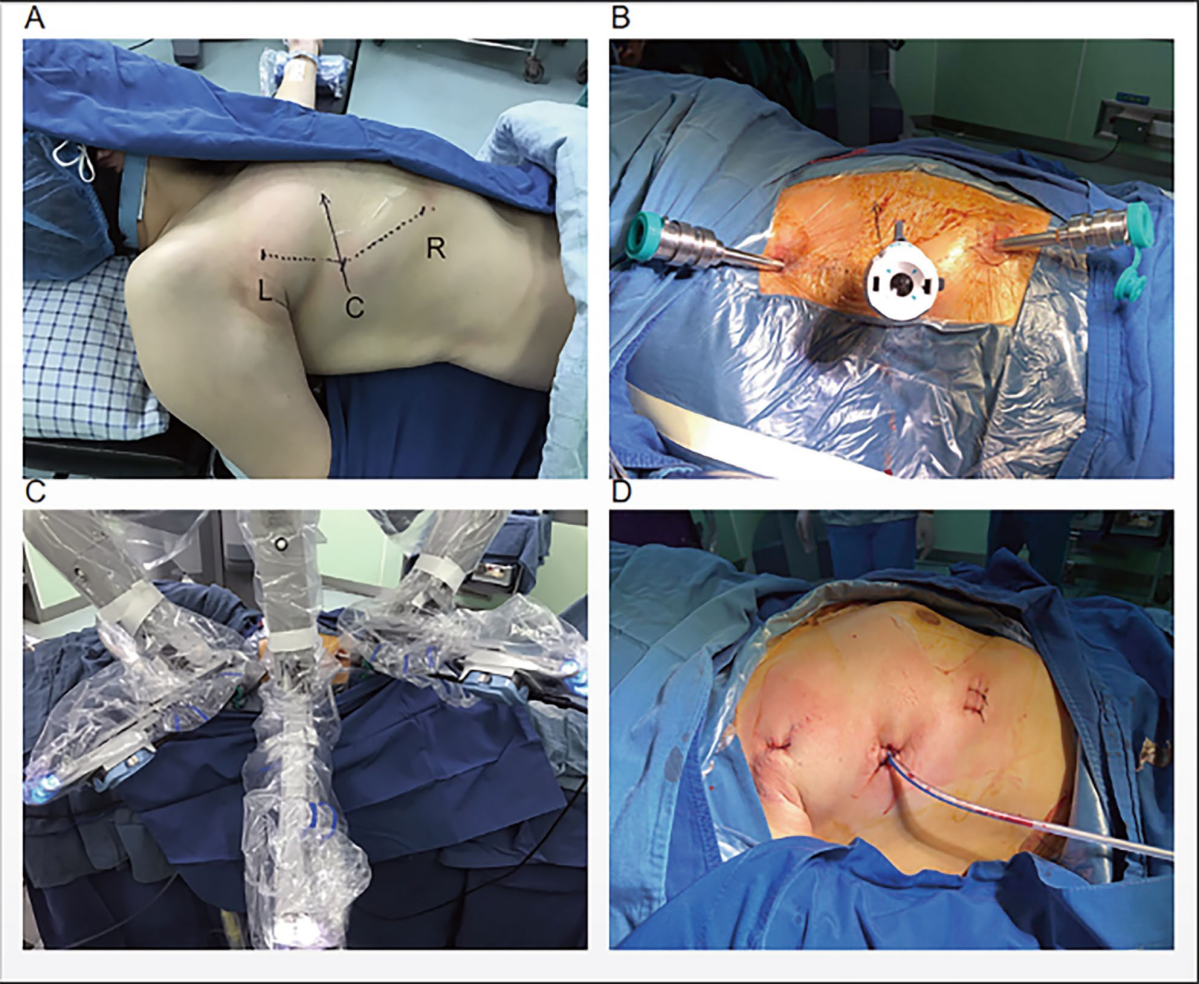
Port positions for robot-assisted thoracic surgery (RATS)

**Fig. 2:**
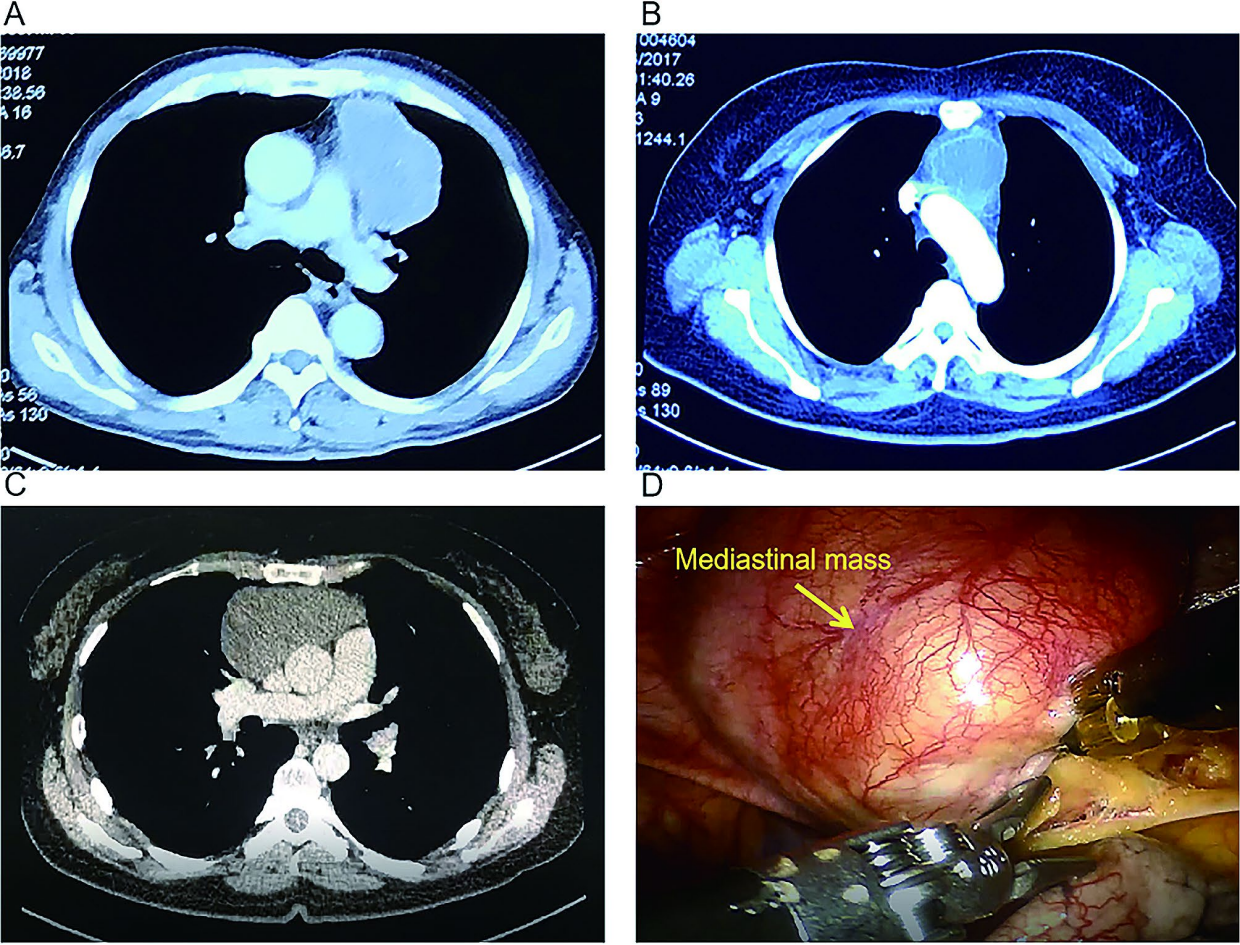
(**A**, **B**) Representative computed tomography images of 2 patients with anterior mediastinal lesion. (**C**, **D**) A 31-year-old woman underwent robot-assisted thoracic surgery on December 16, 2019

**Fig. 3:**
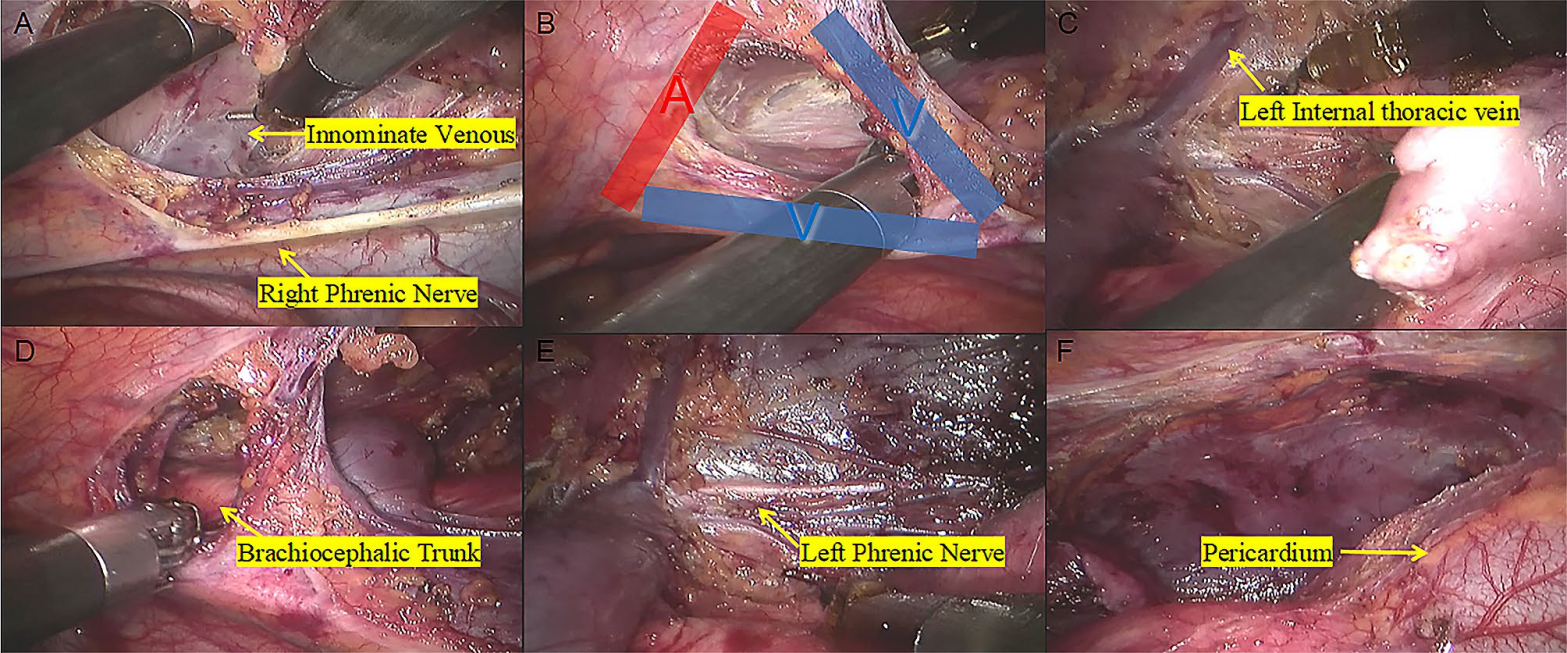
**A**, The innominate vein was carefully dissected just above the right phrenic nerve. **B**, When dissect the thymus superior, keep the anatomical triangle of the Internal thoracic vein, Internal thoracic artery and Superior Vena Cava as far as possible, and conduct the grips to dissect the thymus superior through this area. **C**, The Left Internal thoracic vein was carefully dissected. **D**, Sweep the thymus and reduce misdamage in Brachiocephalic Trunk. **E**, The contralateral phrenic nerve was carefully dissected. **F**, Remove all of the pericardial surface fat

**Fig. 4:**
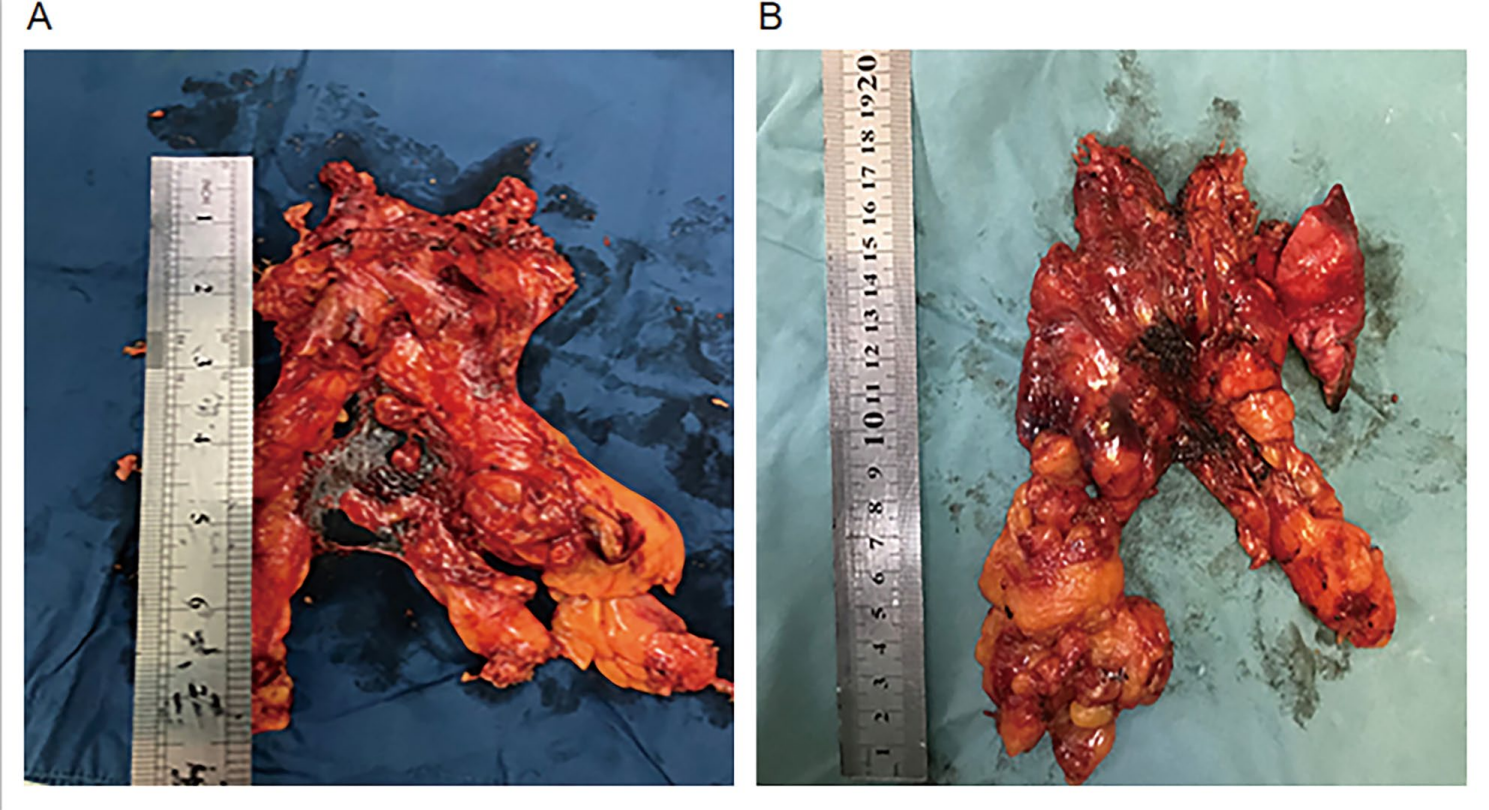
(**A**, **B**) Specimens harvested from AML patients after RATS

During the operation, the three-port technique was utilized, with the ports positioned as illustrated in Fig. 1. A 12 mm camera port was then inserted in the 5th intercostal space (ICS) at the anterior axillary line, followed by CO2 insufflation. Two 8 mm ports were inserted in the 3rd ICS at the anterior axillary line and in the 6th ICS at the middle axillary line, respectively. The grasping arm was inserted into the first port, and the dissecting arm was inserted into the second port. There are some representative chest computed tomography and intraoperative images depicting an anterior mediastinal lesion (See Fig. 2). The extent of the surgery encompassed all adipose tissue within the phrenic nerves on both sides. During the dissection of the superior thymus, care was taken to preserve the anatomical triangle formed by the Internal thoracic vein, Internal thoracic artery, and Superior Vena Cava, and dissection of the superior thymus was performed through this area. This method allowed for full exposure of the lower thyroid pole and the superior thymus pole at the root of the neck, enabling a more complete resection of the anterior mediastinal tumor. Additionally, it facilitated clear visualization of the brachiocephalic trunk, left common carotid artery, and trachea to prevent injury. Simultaneously, both sides of the pleura were kept intact to reduce the risk of extensive tumor spread in the contralateral pleural cavity and minimize lung adhesions for potential secondary surgeries (See Fig. 3). The completely resected anterior mediastinal tumor as show in Fig. 4.

### Statistical analysis

Categorical data were presented as values and percentages, while continuous data were expressed as mean ± standard deviation. The data were prospectively recorded using Microsoft Office Excel 2003 and analyzed utilizing SPSS (IBM). The comparison of continuous variables between groups was conducted using either Student’s t-test or Wilcoxon rank-sum test, while the comparison of categorical data was performed using the chi-square test or Fisher’s exact test. A two-sided p-value of < 0.05 was considered to indicate statistical significance.

## Results

### Demographic and clinical characteristics

In the retrospective analysis of clinical data from 179 patients at our hospital, 92 patients underwent RATS, comprising 47 men and 45 women, with a mean age of 50.4 ± 13.5 years. Additionally, 87 patients underwent VATS, including 39 men and 48 women, with a mean age of 51.5 ± 14.2 years. No significant differences were observed between the RATS and VATS-treated patients in terms of age, sex, or body mass index (BMI). Moreover, there were no significant differences in tumor size or pathological type between the two groups. The pathological types of anterior mediastinal lesions in our study included Thymoma, Thymic hyperplasia, Thymic carcinoma, Thymic cyst, and Lymphoma (refer to Table 1).

**Table 1:** Demographic and clinical characteristics

	*RATS (N = 92)*	*VATS(N = 87)*	*P*-value
**Age, y**	50.4 ± 13.5	51.5 ± 14.2	0.951
**Gender**			
Male	47(51.1%)	39(44.8%)	0.455
Female	45(48.9%)	48(55.2%)	
**BMI**			
≤ 25	50(54.3%)	49(56.3%)	0.881
> 25	42(45.7%)	38(43.7%)	
**Basic illness**			
Hypertension	6(6.5%)	7(8.0%)	0.778
Diabetes	6(6.5%)	6(6.9%)	1.000
COPD	3(3.3%)	2(2.3%)	1.000
**Tumor size**	4.4 ± 1.9	4.5 ± 2.2	0.458
Diagnosis			0.879
Thymoma	73(79.3%)	66(75.9%)	
Thymic hyperplasia	4(4.3%)	9(10.3%)	
Thymic carcinoma	12(13.1%)	7(8.0%)	
Thymic cyst	2(2.2%)	4(4.6%)	
Lymphoma	1(1.1%)	1(1.1%)	

BMI, Body mass index COPD; chronic obstructive pulmonary disease

RATS; Robot-assisted thoracic surgery VATS; video-assisted thoracic surgery

### Surgical related data

As depicted in Table 2, all patients underwent R0 resection, and the total operative time was comparable between the two groups (47.1 ± 9.6 vs. 51.3 ± 12.7 min, *p* = 0.218). In comparison to the VATS group, the RATS group experienced significantly less intraoperative blood loss (79.5 ± 39.6 vs. 97.4 ± 35.8 ml, *p* = 0.042). The volume of drainage did not exhibit a significant difference between the two groups (125.2 ± 34.5 vs. 128.8 ± 40.2, *p* = 0.644). There were no significant differences in the duration of the chest tube between RATS and VATS. RATS patients had shorter postoperative hospital stays compared with VATS patients (2.3 ± 1.0 vs. 3.4 ± 1.4 min, *p* = 0.035). Detailed complications, including fever, injury of the innominate vein, chest pain, pulmonary infection, wound infection, atelectasis, injury of the recurrent laryngeal nerve, and injury of the phrenic nerve, were similar between the two groups.

**Table 2:** Surgical related data

	*RATS(N = 92)*	*VATS (N = 87)*	*P* value	
**Total-operative time (min)**	47.1 ± 9.6	51.3 ± 12.7	0.218	
**Intraoperative blood loss (ml)**	75.9 ± 39.6	97.4 ± 35.8	0.042	
**Volume of drainage (ml)**	125.2 ± 34.5	128.8 ± 40.2	0.645	
**Duration of chest tube (days)**	1.99 ± 0.9	2.16 ± 0.75	0.187	
**Postoperative hospital Stay (days)**	2.3 ± 1.0	3.4 ± 1.4	0.035	
**Intraoperative Adverse events**				
Conversion	0	1(1.1%)	0.486	
Mortality	0	0		
**Complications**				
Fever	6(6.5%)	7(8.0%)	0.778	
Injury of innominate vein	11(11.9%)	8(9.2%)	0.631	
Chest pain	13(14.1%)	16(18.4%)	0.543	
Pulmonary infection	5(5.4%)	7(8.0%)	0.559	
Wound infection	5(5.4%)	5(5.7%)	1.000	
Atelectasis	3(3.3%)	3(3.4%)	1.000	
Injury of phrenic nerve	1(1.1%)	2(2.3%)	0.613	

RATS; Robot-assisted thoracic surgery VATS; video-assisted thoracic surgery

## Discussion

The prevalence of anterior mediastinal lesions is on the rise, primarily encompassing mediastinal goiter and thymoma [1–3, 11–13]. While some cases may not necessitate intervention, the potential for malignant tumors requires adherence to oncological principles, often calling for surgical intervention to enhance prognosis [14]. In recent years, minimally invasive techniques have garnered attention in the surgical management of anterior mediastinal lesions, as they ensure complete tumor resection without the drawbacks of open surgery. Notably, video-assisted thoracoscopic surgery (VATS) has gained traction due to its perceived advantages, including low morbidity, shortened hospitalization, and comprehensive resection [4, 15–17]. However, the advent of robot-assisted technology has introduced a less invasive surgical approach with a broader operative field, enabling access to every aspect of the anterior mediastinum. Initially employed in cardiac surgery, robots were primarily utilized for procedures such as mitral valve repair, atrial fibrillation ablation, left ventricular bipolar pacing lead placement, and atrial septal defect repair [18, 19]. With the rich surgical experience of operators, some scholars have found that compared with video-assisted thoracoscopy, robotics has a steep learning curve, and with the increase of the number of operations, the operation time is also reducing [20, 21]. It is also gradually applied to other thoracic operations. Although, robot-assisted thoracoscopic surgery for anterior mediastinal lesions have been recognized as feasible and safe [20, 22–24]. But no consensus on the surgical method of AML.

The three-port inflation robot-assisted thoracoscopic surgery for mediastinal tumor resection primarily employs a lateral approach, facilitating artificial pneumothorax establishment in the affected side’s pleural cavity. This technique broadens the field of vision, aids in lung collapse, and exposes crucial structures such as the phrenic nerve, thymus vein, and innominate vein, thereby facilitating surgery. Typically, a right-side approach is adopted. Traditional thoracoscopy encounters challenges in thymoma removal and fat dissection, particularly at the contralateral hilum, diaphragmatic angle, neck root, and rear of the innominate vein. This method effectively accomplishes anterior mediastinal fat dissection, including intricate areas of operation.

The robot’s intricate installation process, taking nearly 10 min, results in the actual operation time being shorter for robot-assisted thoracoscopic surgery (RATS) compared to VATS. Intraoperative blood loss was observed to be less in the RATS group than in the VATS group. The robot’s advanced surgical instruments and expanded visual field facilitate superior vessel separation and protection, ultimately reducing intraoperative bleeding. With reduced bleeding, trauma, and correspondingly diminished drainage volume, the duration of chest tube placement is also shortened, leading to expedited patient discharge. Consequently, postoperative hospital stays for patients in the RATS group were notably shorter than those in the VATS group.

One of the common complications of mediastinal surgery is phrenic nerve injury, which can be as high as 7% when using certain energy equipment or when using blunt dissection [25]. By fully utilizing good energy devices and minimizing energy manipulation, it is best to set a sufficient distance between electric coagulation or electric cutting and the nerve to prevent heat damage postoperatively. The right thoracic approach generally displays the right phrenic nerve along the vena cava with minimal variation, whereas the left phrenic nerve can be clearly visualized and protected through the left thoracic approach. When tumors invade the phrenic nerve, it is typically possible to remove it entirely; while attempting to preserve the phrenic nerve as much as possible when the tumor invades it. The robotic anterior mediastinum tumor resection allows for a full, clear, and complete visualization of the phrenic nerve, and effectively reduces the likelihood of phrenic nerve injury. Some researchers have shown that the left-sided approach is better suited to protect the phrenic nerve, while it should be noted that a lack of familiarity with the placement of ports can lead to cardiac injury during left-sided thymectomy [26]. 

Innominate venous injury and bleeding during surgery is possible. When the patient is bleeding, especially from the left subclavian vein, identifying it is difficult. We usually use titanium clips, hom-look or 4 − 0 ETHI-CON barbed thread for continuous suture, and have achieved good results. When the tumor invades surrounding tissues, such as lung, left pericardium, pharyngeal muscle, or vessel, complete resection of the tumor with reduced surgical trauma to the patient can be achieved without an auxiliary hole, and the technique is feasible and reliable.

However, this study has some limitations, such as its retrospective nature and the fact that the technical procedure used depends on the surgeon and patient preferences. The small sample size may lead to selection bias.

In conclusion, this technique for the anterior mediastinum is safe and effective, and it offers the surgeon advice on treatment selection. Overall, the technique has several advantages.

**Legends for illustrations**:

Fig. 1: Port positions for robot-assisted thoracic surgery (RATS). Fig. 2: (A, B) Representative computed tomography images of 2 patients with anterior mediastinal lesion. (C, D) A 31-year-old woman underwent robot-assisted thoracic surgery on December16, 2019. Fig. 3: A, The innominate vein was carefully dissected just above the right phrenic nerve. B, When dissect the thymus superior, keep the anatomical triangle of the Internal thoracic vein, Internal thoracic artery and Superior Vena Cava as far as possible, and conduct the grips to dissect the thymus superior through this area. C, The Left Internal thoracic vein was carefully dissected. D, Sweep the thymus and reduce misdamage in Brachiocephalic Trunk. E, The contralateral phrenic nerve was carefully dissected. F, Remove all of the pericardial surface fat. Fig. 4: (A, B) Specimens harvested from AML patients after RATS.

### Electronic supplementary material

Below is the link to the electronic supplementary material.

Supplementary Material 1

## Data Availability

No datasets were generated or analysed during the current study.
